# CDC’s Hospital-Onset *Clostridioides difficile* Prevention Framework in a Regional Hospital Network

**DOI:** 10.1001/jamanetworkopen.2024.3846

**Published:** 2024-03-27

**Authors:** Nicholas A. Turner, Jay Krishnan, Alicia Nelson, Christopher R. Polage, Ronda L. Sinkowitz-Cochran, Lucy Fike, David T. Kuhar, Preeta K. Kutty, Rachel L. Snyder, Deverick J. Anderson

**Affiliations:** 1 Division of Infectious Diseases, Duke University Medical Center, Durham, North Carolina; 2 Duke Center for Antimicrobial Stewardship and Infection Prevention, Durham, North Carolina; 3 Duke Clinical Microbiology Laboratory, Durham, North Carolina; 4 Centers for Disease Control and Prevention, Atlanta, Georgia

## Abstract

**Importance:**

Despite modest reductions in the incidence of hospital-onset *Clostridioides difficile* infection (HO-CDI), CDI remains a leading cause of health care–associated infection. As no single intervention has proven highly effective on its own, a multifaceted approach to controlling HO-CDI is needed.

**Objective:**

To assess the effectiveness of the Centers for Disease Control and Prevention’s Strategies to Prevent *Clostridioides difficile* Infection in Acute Care Facilities Framework (hereafter, the Framework) in reducing HO-CDI incidence.

**Design, Setting, and Participants:**

This quality improvement study was performed within the Duke Infection Control Outreach Network from July 1, 2019, through March 31, 2022. In all, 20 hospitals in the network participated in an implementation study of the Framework recommendations, and 26 hospitals did not participate and served as controls. The Framework has 39 discrete intervention categories organized into 5 focal areas for CDI prevention: (1) isolation and contact precautions, (2) CDI confirmation, (3) environmental cleaning, (4) infrastructure development, and (5) antimicrobial stewardship engagement.

**Exposures:**

Monthly teleconferences supporting Framework implementation for the participating hospitals.

**Main Outcomes and Measures:**

Primary outcomes were HO-CDI incidence trends at participating hospitals compared with controls and postintervention HO-CDI incidence at intervention sites compared with rates during the 24 months before the intervention.

**Results:**

The study sample included a total of 2184 HO-CDI cases and 7 269 429 patient-days. In the intervention cohort of 20 participating hospitals, there were 1403 HO-CDI cases and 3 513 755 patient-days, with a median (IQR) HO-CDI incidence of 2.8 (2.0-4.3) cases per 10 000 patient-days. The first analysis included an additional 3 755 674 patient-days and 781 HO-CDI cases among the 26 controls, with a median (IQR) HO-CDI incidence of 1.1 (0.7-2.7) case per 10 000 patient-days. The second analysis included an additional 2 538 874 patient-days and 1751 HO-CDI cases, with a median (IQR) HO-CDI incidence of 5.9 (2.7-8.9) cases per 10 000 patient-days, from participating hospitals 24 months before the intervention. In the first analysis, intervention sites had a steeper decline in HO-CDI incidence over time relative to controls (yearly incidence rate ratio [IRR], 0.79 [95% CI, 0.67-0.94]; *P* = .01), but the decline was not temporally associated with study participation. In the second analysis, HO-CDI incidence was declining in participating hospitals before the intervention, and the rate of decline did not change during the intervention. The degree to which hospitals implemented the Framework was associated with steeper declines in HO-CDI incidence (yearly IRR, 0.95 [95% CI, 0.90-0.99]; *P* = .03).

**Conclusions and Relevance:**

In this quality improvement study of a regional hospital network, implementation of the Framework was not temporally associated with declining HO-CDI incidence. Further study of the effectiveness of multimodal prevention measures for controlling HO-CDI is warranted.

## Key Points

**Question:**

Was implementation of the Centers for Disease Control and Prevention’s Strategies to Prevent *Clostridioides difficile* Infection in Acute Care Facilities Framework (hereafter, the Framework) in a regional hospital network associated with a decrease in hospital-onset *C difficile* infections (HO-CDI)?

**Findings:**

In this quality improvement study of 2184 HO-CDI cases (7 269 429 patient-days), the 20 hospitals participating in the Framework had a steeper decline in HO-CDI incidence vs 26 nonparticipating hospitals, but implementation of the Framework was not temporally associated with the decline. The incidence of HO-CDI was declining in participating hospitals before the intervention, and the rate of decline did not change during the intervention.

**Meaning:**

Findings of this study suggest that benefits from implementation of the Framework warrant further study.

## Teaser

This quality improvement study investigates whether implementation of the Centers for Disease Control and Prevention’s Strategies to Prevent *Clostridioides difficile* Infection in Acute Care Facilities Framework is associated with a decrease in hospital-onset *C difficile* infections.

## Introduction

Hospital-onset *Clostridioides difficile* infection (HO-CDI) remains a leading cause of health care–associated infection in the US, with an estimated incidence of 8.3 cases per 10 000 patient-days.^1,2^ Prevention efforts have only modestly decreased CDI incidence.^3,4^ Hospitals need feasible and effective HO-CDI prevention strategies, especially as CDI-related health care costs, morbidity, and mortality remain substantial: in the US, CDI was responsible for over $1 billion in health care costs and nearly 13 000 deaths in 2017 alone.^5^

The Centers for Disease Control and Prevention created the Strategies to Prevent *Clostridioides difficile* Infection in Acute Care Facilities Framework on November 23, 2018 (hereafter, the Framework) to codify evidence-based HO-CDI prevention strategies for acute care settings.^6,7^ The Framework has 39 discrete intervention categories organized into 5 focal areas for CDI prevention: (1) isolation and contact precautions, (2) CDI confirmation, (3) environmental cleaning, (4) infrastructure development, and (5) antimicrobial stewardship engagement.^6^ Although individual recommendations within the Framework are evidence-based,^8,9^ to our knowledge, there are no clinical studies assessing the effectiveness of the Framework as a whole.

In this multicenter, longitudinal quality improvement study, we assessed the Framework’s effectiveness within a network of 20 regional hospitals. The primary outcome was HO-CDI incidence over time.

## Methods

### Study Design and Outcomes

We conducted a longitudinal quality improvement study of HO-CDI incidence within the Duke Infection Control Outreach Network. Hospitals were invited to participate if their HO-CDI incidence rate was above the network’s median prior to study launch. Participating hospitals were guided through the Framework implementation from July 1, 2019, through March 31, 2022. The primary outcome—assessing the Framework’s association with HO-CDI incidence—was evaluated through 2 analyses. First, temporal trends in HO-CDI incidence were compared between intervention sites (20 hospitals participating in the Framework implementation) and control sites (26 hospitals that submitted HO-CDI data but did not participate in the Framework implementation). Second, we compared trends within the 20 participating hospitals before and after implementation using additional HO-CDI incidence data from July 1, 2017, through June 30, 2019, as a preintervention period.

Secondary outcomes—including assessment of the implementation’s dose-dependent association and the association of individual Framework measures with HO-CDI incidence—were evaluated among the 20 intervention sites from July 1, 2019, through March 31, 2022. Study design was reviewed by the Duke University Health System Institutional Review Board and was deemed exempt, with a waiver of informed patient consent granted for data collection, as the study did not involve direct intervention or interaction with patients. This study followed the Standards for Quality Improvement Reporting Excellence (SQUIRE) reporting guideline.

### Implementation of HO-CDI Prevention Strategies

To support Framework implementation, infection preventionists from each intervention hospital participated in an in-person launch event followed by monthly teleconference calls. Each call began with a focused presentation on a Framework strategy followed by open discussion. Throughout the study period, the central study team prepared and released 42 tools designed to support Framework implementation. A complete listing of tools is provided in eTable 1 in Supplement 1 and online (eAppendix 2 in Supplement 1), including sample outputs of tools that were developed as a dashboard, report, or web application. The central study team also prepared and distributed quarterly summaries of each site’s root cause analyses (example report included as eAppendix 1 in Supplement 1). While adoption of prevention measures across all Framework areas was strongly encouraged, each hospital exercised independent decision-making regarding which measures to implement, when implementation would occur, and how measures would be enacted.

### Data Collection and Definitions

*C difficile* infections were defined in accordance with the National Healthcare Safety Network LabID definition: cases were identified by a positive *C difficile* laboratory test result by either toxin A/B assay or nucleic acid amplification testing from stool samples submitted to each hospital’s clinical microbiology laboratories.^10^ We define HO-CDI cases as those occurring after hospital day 3. Any repeat samples positive for *C difficile* that were sent within 14 days of a prior positive sample were considered to be duplicates and were not counted separately. Cases of HO-CDI and patient-days present were electronically collected and submitted by infection preventionists at each participating hospital. Incidence rates of HO-CDI were calculated monthly per 10 000 patient-days.

Framework implementation was tracked through quarterly surveys completed by infection preventionists at each participating site indicating whether any additional Framework measures were enacted in the interval since the last survey and, if so, when the change was enacted. Sites were assigned a monthly intervention score, indicating how many Framework interventions out of 39 possible were in effect.

### Statistical Analysis

We used generalized estimating equation models for regression analysis of primary and secondary outcomes to account for the violation of independence inherent in repeated measures data clustered by hospital. Primary analyses were conducted by intention-to-treat principles; for example, associations with the Framework implementations were measured over the study period regardless of degree of implementation. Separate models were constructed for each analysis. The first analysis compared HO-CDI trends between intervention and control sites using variables for time, arm (intervention vs control), and an interaction term (time × arm) to test for the slope difference between intervention and control hospitals. The second analysis used an interrupted time-series construct to compare HO-CDI trends before and after Framework implementation: a baseline time variable was used to adjust for preintervention HO-CDI trends; an intervention term marking study onset assessed for level changes in HO-CDI incidence; and a time-since-study-onset term was used to assess for postintervention changes in HO-CDI incidence.^11^ Various lag times from 0 to 3 months were assessed, but did not significantly alter model performance or parameter estimates. Results are presented with a 1-month lag as a pragmatic interval over which interventions might be expected to take effect. As this study consisted of a convenience sample of hospitals amenable to participation and there are no widely accepted conventions for longitudinal power calculations, a priori power calculations were not conducted.

For all secondary outcomes, Framework interventions were modeled as time-varying covariates in generalized estimating equation models. Interventions were assessed using the structure described by Wagner et al^11^ with level associations modeled by dummy variables indicating whether a prevention measure was used during a given month and slope effects modeled using time-since-study-onset variables. To account for differences in Framework measure adoption between sites and over time, secondary analyses were conducted by a per-protocol principle. Outcomes were assessed relative to Framework intervention implementation rather than merely study participation.

Data were analyzed from April 2022 through June 2023. All statistical models were constructed using geepack or glmmTMB packages in R, version 4.1.2 (R Project for Statistical Computing). All graphs were created using the ggplot2 package. Data were assessed for evidence of zero inflation and overdispersion. Inspection of quantile-quantile plots suggested the Poisson distribution to be a reasonable assumption in each case. Fitted vs actual plots were constructed as a final visual inspection of each model’s accuracy.

Statistical significance for primary and secondary outcomes was assessed from the relevant time × intervention interaction or time-since-study-onset term (reflecting HO-CDI incidence trends). Throughout this study, a significance threshold of *P* < .05 was used; 95% CIs were calculated by the Wald method. The incidence rate ratio (IRR) was reported as a measure for comparing change in outcome counts.

Because of the inherent complexity of time-series data, we repeated each analysis using mixed-effects modeling to assess model stability across varying correlation structures. Mixed-effects models are an alternative to generalized estimating equations that adjust for potential clustering (hospital-level correlations) through the use of random effects.^12^ Additionally, we conducted a post hoc sensitivity analysis incorporating COVID-19 pandemic effects into each time-series model using March 2020 as a cutoff to delineate prepandemic vs pandemic time periods.

## Results

The intervention cohort included 20 Southeastern US hospitals within the Duke Infection Control Outreach Network, with a median (IQR) capacity of 226 (161-282) beds; the control cohort included 26 Southeastern US hospitals with a median (IQR) capacity of 193 (121-311) beds (Table 1). The study sample included a total of 2184 HO-CDI cases and 7 269 429 patient-days. The intervention cohort included 3 513 755 patient-days and 1403 HO-CDI cases from July 2019 through March 2022 (median [IQR] HO-CDI incidence rate, 1.1 [0.7-2.7] cases per 10 000 patient-days). For the first analysis comparing intervention hospitals with control hospitals, the control cohort included an additional 3 755 674 patient-days and 781 HO-CDI cases from July 2019 through March 2022 (median [IQR] HO-CDI incidence rate, 1.1 [0.7-2.7] cases per 10 000 patient-days). For the second analysis assessing HO-CDI incidence trends within intervention hospitals, the 24-month preintervention period captured an additional 2 538 874 patient-days and 1751 HO-CDI cases (median [IQR] HO-CDI incidence rate, 5.9 [2.7-8.9] cases per 10 000 patient-days).

**Table 1.** Hospital Characteristics Prior to Framework Implementation Study Onset

Characteristic	Intervention hospital (n = 20)	Control hospital (n = 26)
Bed capacity, median (IQR)	226 (161-282)	193 (121-311)
Intensive care unit beds, median (IQR)	16 (11-28)	13 (8-33)
Annual admissions, median (IQR)	10 323 (7560-14 633)	8106 (3698-10 731)
Teaching hospital, No. (%)	15 (75)	13 (50)
Hospital location, No. (%)		
Florida	1 (5)	1 (4)
Georgia	0	15 (58)
North Carolina	15 (75)	8 (31)
South Carolina	0	1 (4)
Virginia	3 (15)	1 (4)
West Virginia	1 (5)	0
HO-CDI incidence per 10 000 patient-days, median (IQR)	2.8 (2.0-4.3)	1.1 (0.7-2.7)

Abbreviation: HO-CDI, hospital-onset *Clostridioides difficile* infection.

The Framework’s 39 discrete interventions across 5 categories (Table 2) were used to calculate an intervention score each month for each site participating in the intervention. Participating sites had a median (IQR) of 14 (13-15) Framework interventions in place at baseline and a median (IQR) of 19 (17-20) Framework interventions at the end of the study. Isolation until at least 48 hours after resolution of diarrhea, hand hygiene auditing, laboratory rejection of formed stool, and the use of sporicidal cleaning agents were universally used at study onset (Table 2). Strategies frequently selected for improvement included isolation practice audits, case reviews to identify areas for improvement, and 2-step testing. As hospitals varied in Framework measures implemented at baseline and over time, eTable 2 in Supplement 1 summarizes Framework implementation relative to opportunity gaps, and eFigure 1 in Supplement 1 shows how many Framework measures were implemented over time.

**Table 2.** Framework Interventions Implemented at Baseline and at the End of Study

Intervention	Intervention, No. (%)		
	Present at baseline (n = 20)	Present at end of study (n = 20)	Increase, No./total No. (%)^a^
Isolation and contact precautions			
Nurse-driven rapid isolation	19 (95)	19 (95)	0
Isolation until 48 h after resolution of diarrhea	20 (100)	20 (100)	0
Isolation for duration of hospitalization	18 (90)	18 (90)	0
Improved isolation during unit transfer	1 (5)	1 (5)	0
Single-use equipment	13 (65)	13 (65)	0
Isolation practices auditing	0	7 (35)	7/20 (35)
Other^b^	1 (5)	5 (25)	4/19 (21)
Infrastructure development			
Hand hygiene education	0	1 (5)	1/20 (5)
Hand hygiene audit improvement	0	3 (15)	3/20 (15)
Hand hygiene auditing frequency	0	2 (10)	2/20 (10)
Hand hygiene protocol	20 (100)	20 (100)	0
Hand hygiene audit initiation	20 (100)	20 (100)	0
Infrastructure workgroup	0	1 (5)	1/20 (5)
Infrastructure education	0	0	0/20
Case reviews to identify areas for improvement	2 (10)	6 (30)	4/18 (22)
Other infrastructure-related intervention^c^	0	5 (25)	5/20 (25)
Other hand hygiene–related intervention^d^	0	4 (20)	4/20 (20)
CDI confirmation			
Avoiding repeat *C difficile* testing	14 (70)	15 (75)	1/6 (17)
Avoiding test of cure	0	0	0
Considering alternative diagnoses	13 (65)	13 (65)	0
Avoiding testing while patient taking laxatives	15 (75)	15 (75)	0
Laboratory rejection of formed stool	20 (100)	20 (100)	0
Change in laboratory reporting	0	1 (5)	1/20 (5)
2-Step testing	2 (10)	10 (50)	8/18 (44)
Other clinical intervention^e^	0	11 (55)	11/20 (55)
Other laboratory intervention^f^	0	4 (20)	4/20 (20)
Environmental cleaning			
UV light	12 (60)	13 (65)	1/8 (13)
Cleaning audits	10 (50)	13 (65)	3/10 (30)
Cleaning additional patient care areas	11 (55)	11 (55)	0
Use of sporicidal cleaning agents	20 (100)	20 (100)	0
Daily cleaning protocols	18 (90)	19 (95)	1/2 (50)
Terminal cleaning protocols	7 (35)	10 (50)	3/13 (23)
Other^g^	2 (10)	7 (35)	5/18 (28)
Antimicrobial stewardship engagement			
Institution-specific treatment guidelines	5 (25)	5 (25)	0
Targeting improved duration of antibiotic treatment	1 (5)	3 (15)	2/19 (11)
Targeting antibiotics with a high risk of CDI	0	4 (20)	4/20 (20)
Fluoroquinolone restriction	0	2 (10)	2/20 (10)
Focus on duration of antibiotic treatment at discharge	1 (5)	1 (5)	0
Other^h^	0	13 (65)	13/20 (65)

Abbreviation: *C difficile, Clostridioides difficile*; CDI, *C difficile* infection; Framework, Centers for Disease Control and Prevention’s Strategies to Prevent *Clostridioides difficile* Infection in Acute Care Facilities Framework; HO-CDI, hospital-onset *C difficile* infection.

^a^ Percentage increase calculated as proportion of facilities enacting change within a given Framework area divided by the number of facilities not yet reporting the given practice at baseline.

^b^ Examples include inclusion of contact precaution training in nursing orientation, updates to isolation carts or caddies, and education of visitors on isolation measures.

^c^ Examples include change in clinical leadership, change in structure of committees monitoring HO-CDI rates, and addition of HO-CDI rate discussions to nursing huddles.

^d^ Examples include creating a formal auditing team, requiring unit managers to monitor hand hygiene, changing the hand hygiene monitoring app or tool, and changing the number of audits conducted monthly.

^e^ Examples include limiting *C difficile* testing to day shift personnel, providing clinician and nurse education, assessing for alternative causes of diarrhea, and updating hospital orders to remind staff to submit only unformed specimens.

^f^ Examples include altering laboratory protocol for specimen review and rejection if specimen was unformed, and changing the wording of laboratory reports for 2-step testing results.

^g^ Examples include expanding staff education and adenosine triphosphate audits.

^h^ Examples include adding pharmacist to *C difficile* case review team and updating antibiotic ordersets.

### Primary Outcome

**Figure 1.**
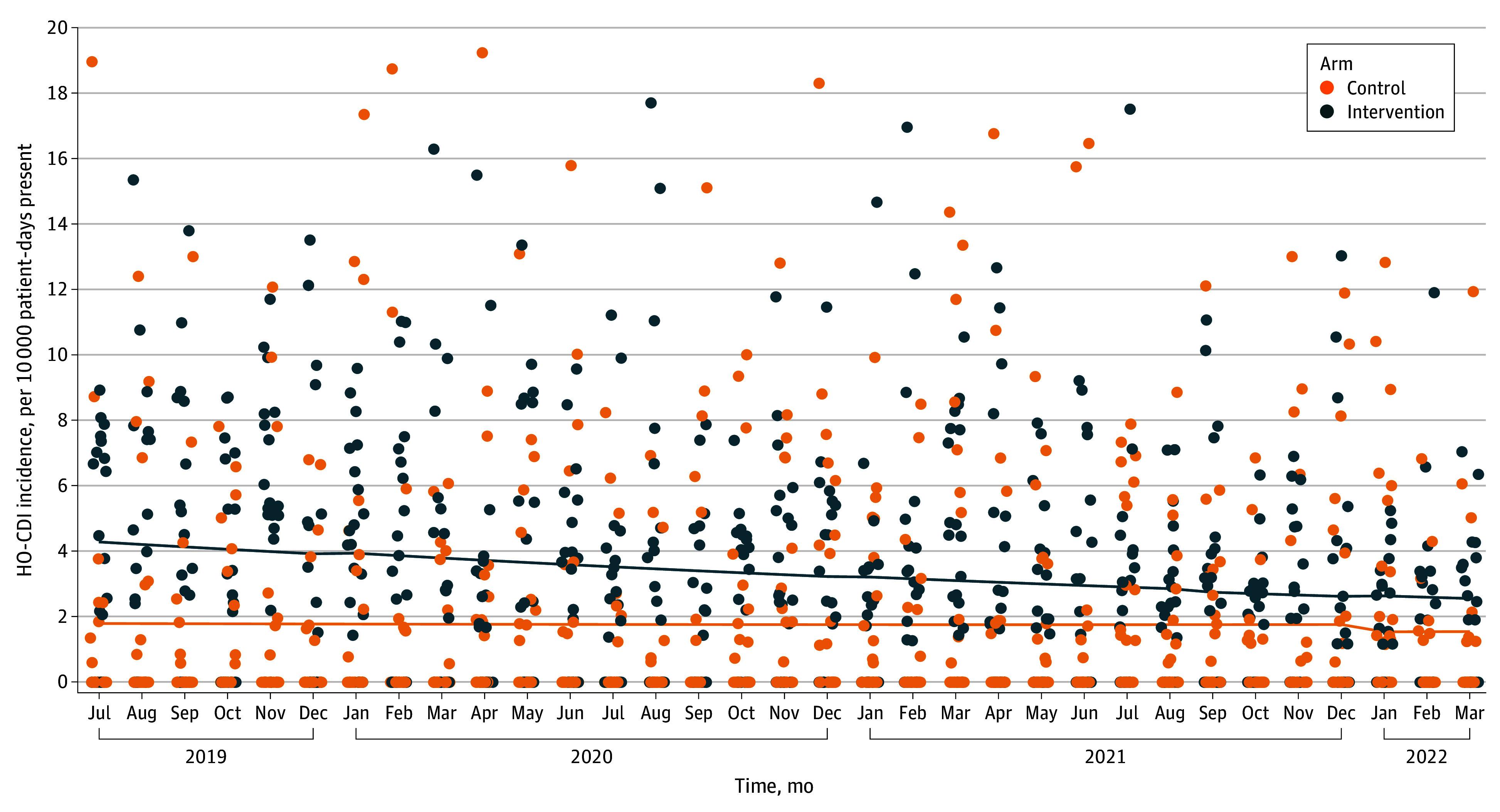
Comparison of Hospital-Onset *Clostridioides difficile* Infection (HO-CDI) Incidence Trends Between Participating Hospitals and Control Hospitals The solid lines represent the preintervention trend.

Comparison of trends in HO-CDI incidence between intervention and control hospitals revealed that intervention hospitals had a higher rate of HO-CDI from the outset (yearly IRR, 2.79; 95% CI, 1.10-7.05). For the first analysis, we observed a modest but nonsignificant decline in HO-CDI incidence with time, and a steeper decline in HO-CDI incidence among intervention hospitals relative to controls (yearly IRR, 0.79 [95% CI, 0.67-0.94]; *P* = .01) (Figure 1). The steeper decline in HO-CDI incidence observed among intervention hospitals remained consistent even after adjustment for the COVID-19 pandemic and varying statistical model specifications and structures (eTables 3 and 4 in Supplement 1).

**Figure 2.**
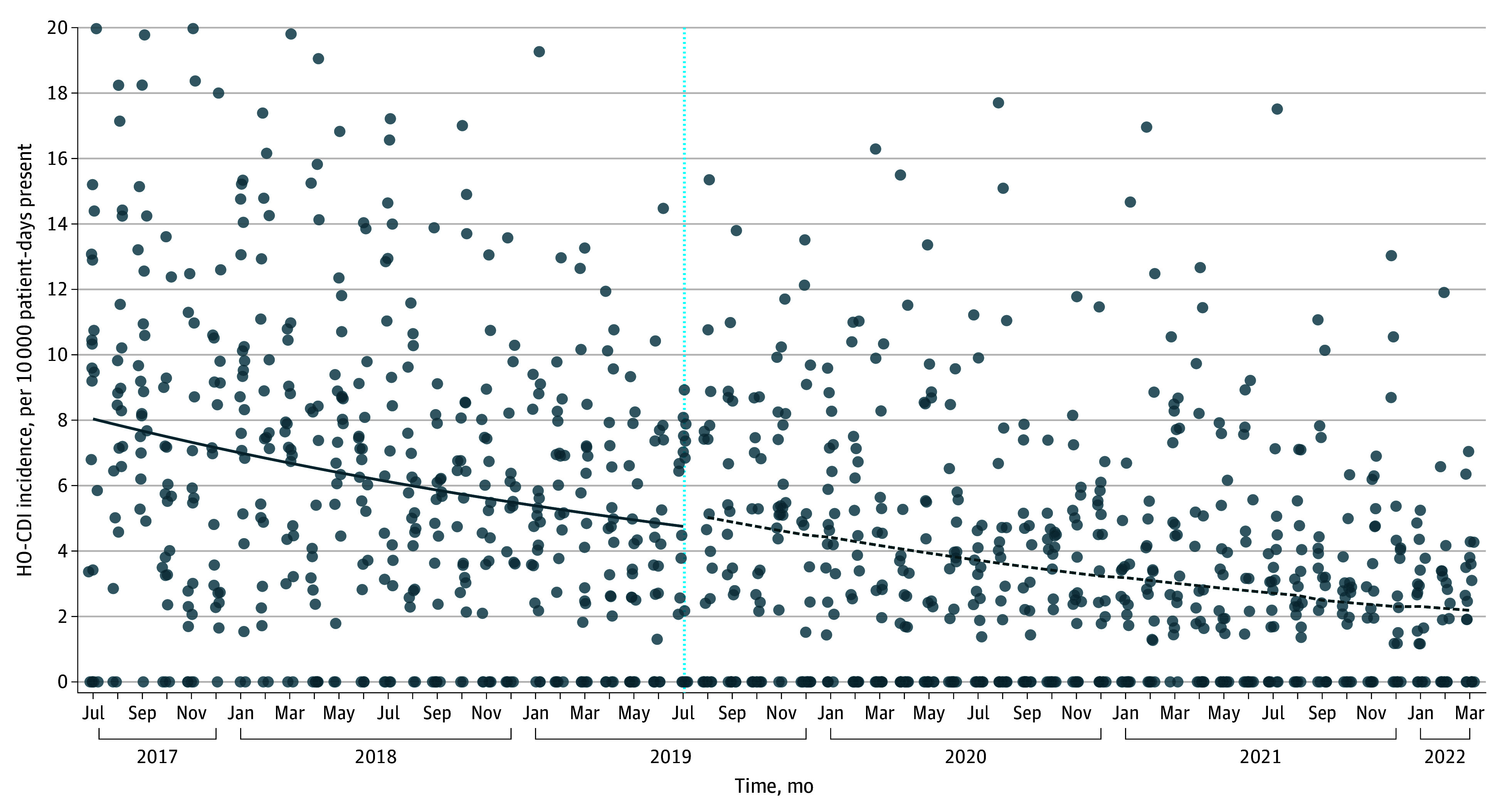
Hospital-Onset *Clostridioides difficile* Infection (HO-CDI) Incidence Trends Among Participating Hospitals 24 Months Before and After Study Participation The vertical line represents the start of Framework implementation; the solid line represents the preintervention trend; the dashed line represents the postintervention trend.

For the second analysis comparing HO-CDI incidence trends before and after intervention within participating sites, we observed a significant preintervention decline in HO-CDI incidence (yearly IRR, 0.76; 95% CI, 0.68-0.85]; *P* < .01). However, we did not observe a significant change in HO-CDI incidence temporally associated with study onset (yearly IRR, 0.98 [95% CI, 0.77-1.24]; *P* = .85) (Figure 2). Effect estimates remained stable even after accounting for the COVID-19 pandemic and multiple statistical model structures (eTables 5-7 in Supplement 1). Because primary end points were not jointly met, the overall primary end point was not met.^13^

### Secondary Outcomes

To account for differences in Framework measure adoption between sites and over time, secondary analyses were conducted by a per-protocol principle. Outcomes were assessed relative to Framework intervention implementation rather than merely study participation.

**Figure 3.**
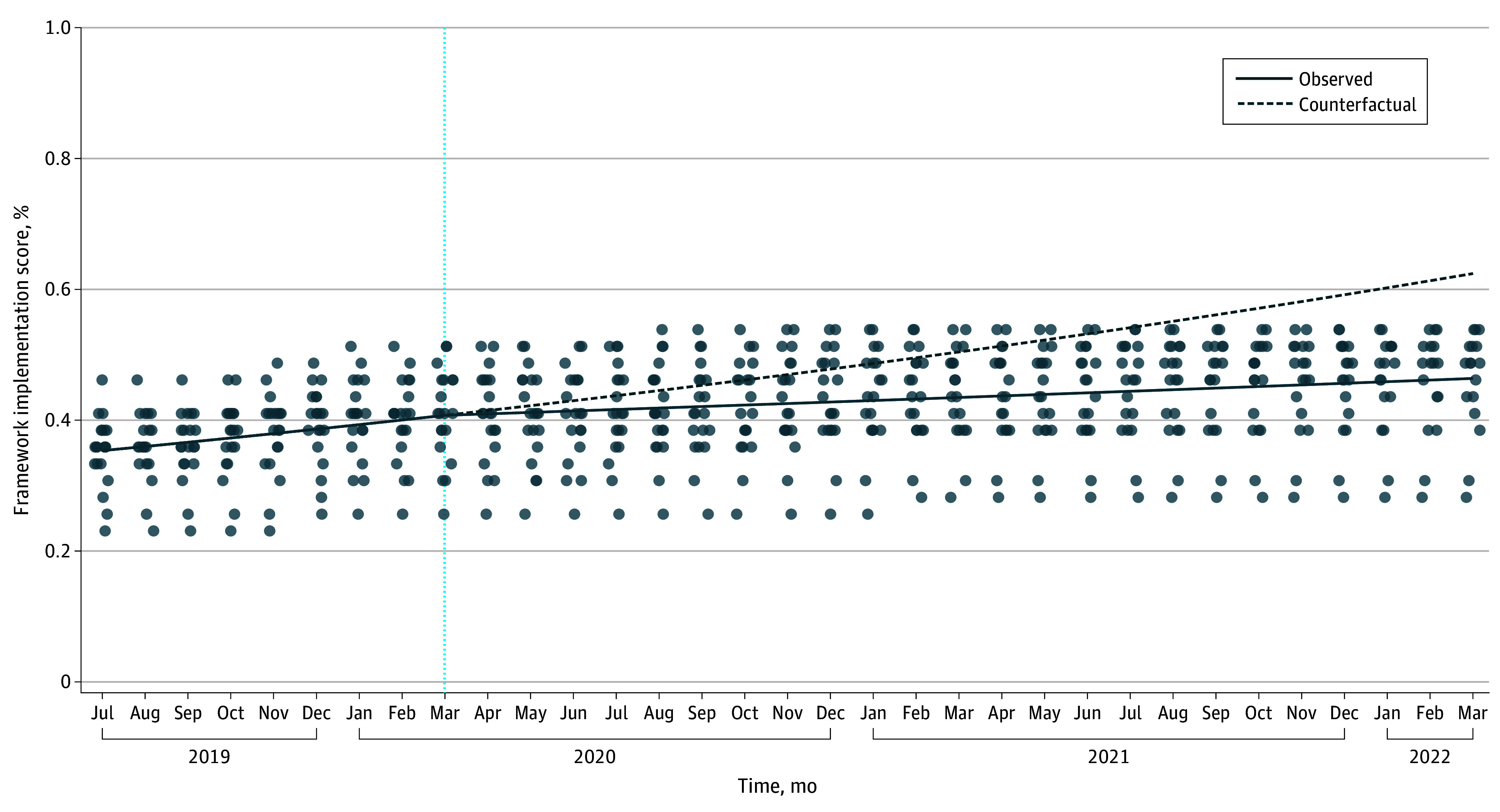
Association of COVID-19 Pandemic With Rate of Implementation of the Framework The counterfactual line represents projected Framework intervention scores if initial pre–COVID-19 trends had been sustained throughout the study period (eg, using only the prepandemic model slope to estimate interventions through study close). The vertical dashed line represents the beginning of the COVID-19 pandemic.

The selection and timing of Framework measure implementation varied between intervention sites. The COVID-19 pandemic also affected Framework implementation. Before the pandemic, Framework intervention scores were increasing by an average of 27% per year (yearly IRR, 1.27; 95% CI, 1.15-1.40). After pandemic onset, the rate of change in Framework intervention scores fell significantly (yearly IRR, 0.84 [95% CI, 0.75-0.94]; *P* = .001), equating to an average postpandemic implementation score increase of only 7% per year (Figure 3).

Assessment by total relative Framework intervention score revealed a similar baseline trend toward reduced HO-CDI incidence with time (yearly IRR, 0.81; 95% CI, 0.68-0.97), but also showed an association between increased total Framework intervention score and rate of decline in HO-CDI incidence (yearly IRR, 0.95 [95% CI, 0.90-0.99]; *P* = .03), equivalent to an estimated 5% decline for each additional Framework measure undertaken (eTable 8 in Supplement 1). Because intervention score effects may not be linear, we conducted a sensitivity analysis whereby sites were ranked in quintiles for the number of Framework measures undertaken over time. We then used a time × intervention quintile term to test for differences in slopes of HO-CDI incidence over time across quintiles, again confirming that sites in higher intervention quintiles exhibited a greater decline in HO-CDI incidence over time, equating to an approximately 11% decline in incidence rate for each step up in Framework implementation quintile (eTable 8 in Supplement 1).

Assessment of the association between individual prevention measures and HO-CDI incidence was hindered by the small number of changes made in context of the COVID-19 pandemic. Consequently, analysis of individual Framework interventions was limited to those undertaken by at least 2 sites for the full model and further restricted to those with at least 6 months of preintervention and postintervention data available for the limited model to improve reliability. Based on estimated 95% CIs, a few interventions appeared to be associated with reduced HO-CDI incidence, including case reviews to identify areas for improvement, 2-step testing, and stewardship interventions focused on antibiotics associated with a high risk of CDI (eTable 9 in Supplement 1).

As a post hoc assessment of the COVID-19 pandemic’s effect on study power, we used prepandemic intervention score trends and the estimated association between intervention scores and HO-CDI incidence to estimate how many additional cases of HO-CDI might have been prevented if prepandemic intervention trends had been maintained. We estimate that lost intervention opportunities could have accounted for as much as an additional 40% relative reduction in HO-CDI incidence by study close (eFigure 2 in Supplement 1).

## Discussion

This quality improvement study found that, while hospitals participating in the Framework implementation had a steeper decline in HO-CDI incidence relative to controls, the time-series analysis revealed that this trend was not temporally associated with study participation. Rather, HO-CDI incidence was already falling across intervention sites prior to study onset. The disparate results between our 2 primary analyses highlights one of the strengths of time-series analysis: the use of external controls or simple before-and-after comparisons may fail to account for preexisting trends that can otherwise inflate type I error rates. In this case, higher HO-CDI incidence among intervention sites might have important implications for interpreting study results. The higher HO-CDI rate among participating hospitals was anticipated since invitation to participate was contingent on having an above-median HO-CDI rate. Regression toward the mean may have contributed to the steeper decline in HO-CDI incidence observed among intervention sites. Alternatively, sites with higher HO-CDI incidence rates may already have been enacting *C difficile* control measures prior to study onset that were not captured. In either case, it remains appropriate to consider that implementation of the Framework was not temporally associated with declining HO-CDI incidence.

Considering the context of the COVID-19 pandemic, however, an entirely different possibility emerges. Rates of Framework implementation fell significantly after the onset of the COVID-19 pandemic, perhaps reflecting the redirection of infection prevention personnel to assist with the COVID-19 response or the high levels of exhaustion experienced by infection prevention personnel during the pandemic.^14^ With sharply dwindling Framework measure implementation rates early in the study period, study power to detect differences in HO-CDI incidence attributable to study interventions was likely severely reduced. Although post hoc power calculations are fraught with difficulty, model projections suggest a greater than 30% reduction in total Framework measures undertaken by the end of study relative to the anticipated number projected from prepandemic trends. Using the incidence rate changes from the second analysis of intervention effects before vs after the pandemic, lost intervention opportunities could have accounted for an additional 40% relative reduction in HO-CDI incidence by study close.

With lower-than-expected Framework implementation rates, secondary analyses that accounted for changes associated with specific Framework measures take on new importance. If a null result is driven primarily by ineffective interventions, then accounting for the degree of implementation should have no implications for the overall outcome. In contrast, if low implementation rates contribute to a null result, then a per-protocol analysis may still inform whether an intervention could have value if it were reliably implemented. After accounting for the degree of Framework implementation, we did detect a dose-dependent association between increased implementation of Framework measures and a steeper decline in HO-CDI incidence.

While the overall number of Framework measures undertaken lagged below expectations in the setting of the pandemic, a few were implemented across enough sites to at least allow an exploratory look at individual associations between prevention measures and HO-CDI incidence. After filtering to include only those interventions undertaken by at least 2 sites with at least 6 months of pre- and postintervention data available, a few interventions appeared to be associated with lower HO-CDI rates. Specifically, case reviews to identify areas for improvement and stewardship interventions designed to target antibiotics with a high risk of CDI were associated with estimated trends toward lower HO-CDI incidence over time. Our secondary analyses are consistent with prior studies: Regarding high-risk antibiotic use, several prior longitudinal modeling studies found similar associations between lower use of high-risk antibiotics and reduced CDI incidence.^15,16^ While the specific root cause analysis tool built for this study has not been previously assessed, most quality improvement efforts have relied on similar structured review tools that have been successfully used to track implementation gaps over time.^17,18^ Also consistent with prior research, conversion to 2-step testing appeared to be associated with a reduction in HO-CDI incidence.^6,19^

To our knowledge, this is the first study to calculate an intervention score derived from the Framework and correlate change in this score over time with HO-CDI incidence. One of the long-standing challenges in infection prevention research stems from the inherent difficulty in promoting uptake of infection prevention measures. Implementation science depends on metrics of practice update to study the gap between theory and practice.^20,21^ If externally validated, use of an intervention score based on the Framework could be a valuable tool for bridging the current gap between HO-CDI prevention recommendations and real-world practice.

### Limitations

While key limitations related to study power and pandemic effect have been discussed, there are other limitations. As with any observational study, unmeasured confounders may remain. Specifically, most sites reported 1 or more HO-CDI prevention interventions other than the 39 covered in the Framework. Because these interventions were heterogeneous, there was no reliable way to account for them in our modeling. Intervention assessment might also have been easier if the timing, order, and implementation of Framework measures were specified in a structured manner across sites. We elected not to be overly prescriptive, however, as this would have greatly increased study cost and complexity while reducing real-world relevance. Additionally, since this study did not capture measures of adherence to Framework measures over time, it is possible that such changes might also have affected HO-CDI incidence trends.

## Conclusions

In this quality improvement study within a Southeastern US regional hospital network, implementation of the Framework was not temporally associated with declining HO-CDI incidence. However, the COVID-19 pandemic was associated with markedly lower Framework implementation rates and thus prevented us from ruling out benefit. Multiple secondary analyses suggested steeper rates of decline in HO-CDI incidence among sites that successfully implemented Framework measures despite the pandemic. A few interventions—case reviews to identify areas for improvement, stewardship interventions targeting antibiotics with a high risk of CDI, and 2-step testing—showed potential benefit despite low anticipated power to detect such benefits. Besides highlighting the need for robust infection prevention infrastructure (capable of dealing with routine duties and the occasional pandemic), the dose-dependent association of Framework measures with the decline in HO-CDI cases suggests that HO-CDI prevention hinges heavily on how effectively Framework measures are implemented. External validation of the effectiveness of a multimodal prevention measure, such as the Framework, for controlling HO-CDI could be useful for future research merging insights from implementation science with clinical studies.

## Notes

Supplement 1. **eTable 1.** HO-CDI Prevention Tools and Interventions Deployed, Indexed by Framework Area **eTable 2.** Overall Relative Changes in Core HO-CDI Prevention Areas by Intervention Site, July 2019-March 2022 **eTable 3.** Time Series Modeling of HO-CDI Incidence Between Intervention Sites and Control Sites With COVID-19 Effect (First Coprimary Analysis), July 2017-March 2022 **eTable 4.** Time Series Modeling of HO-CDI Incidence Between Intervention Sites and Control Sites Across a Range of Statistical Model Specifications and Structures (First Coprimary Analysis), July 2017-March 2022 **eTable 5.** Sensitivity Analysis of Second Coprimary Analysis (Pre- and Postintervention HO-CDI Trends Within Intervention Sites) Accounting for Potential COVID-19 Effect, July 2017-March 2022 **eTable 6.** Sensitivity Analysis of Second Coprimary Analysis (Pre- and Postintervention HO-CDI Trends Within Intervention Sites) Accounting for Varying Statistical Model Structures, July 2019-March 2022 **eTable 7.** Sensitivity Analysis of Second Coprimary Analysis (Pre- and Postintervention HO-CDI Trends Within Intervention Sites) Accounting for Potential COVID-19 Effect, July 2019-March 2022 **eTable 8.** Dose Effect of Framework Intervention Score **eTable 9.** Association of Individual Framework Prevention Measures With HO-CDI Incidence Rate Trends, July 2019-March 2022 **eFigure 1.** Total Framework Intervention Implementation Score Over Time **eFigure 2.** Post Hoc Assessment of Study Power Loss due to COVID-19 **eAppendix 1.** Markdown Files from Statistical Analysis in R **eAppendix 2.** Supplemental Appendix Accessible via Open Science Framework Repository

Supplement 2.
Data Sharing Statement
