# Supplementary material for: CDC’s Hospital-Onset Clostridioides difficile Prevention Framework in a Regional Hospital Network
Source: JAMA Netw Open. 2024 Mar 27;7(3):e243846. doi: 10.1001/jamanetworkopen.2024.3846 (PMC10973890; doi:10.1001/jamanetworkopen.2024.3846)
Supplement: Supplement 1. — eTable 1. HO-CDI Prevention Tools and Interventions Deployed, Indexed by Framework Area eTable 2. Overall Relative Changes in Core HO-CDI Prevention Areas by Intervention Site, July 2019-March 2022 eTable 3. Time Series Modeling of HO-CDI Incidence Between Intervention Sites and Control Sites With COVID-19 Effect (First Coprimary Analysis), July 2017-March 2022 eTable 4. Time Series Modeling of HO-CDI Incidence Between Intervention Sites and Control Sites Across a Range of Statistical Model Specifications and Structures (First Coprimary Analysis), July 2017-March 2022 eTable 5. Sensitivity Analysis of Second Coprimary Analysis (Pre- and Postintervention HO-CDI Trends Within Intervention Sites) Accounting for Potential COVID-19 Effect, July 2017-March 2022 eTable 6. Sensitivity Analysis of Second Coprimary Analysis (Pre- and Postintervention HO-CDI Trends Within Intervention Sites) Accounting for Varying Statistical Model Structures, July 2019-March 2022 eTable 7. Sensitivity Analysis of Second Coprimary Analysis (Pre- and Postintervention HO-CDI Trends Within Intervention Sites) Accounting for Potential COVID-19 Effect, July 2019-March 2022 eTable 8. Dose Effect of Framework Intervention Score eTable 9. Association of Individual Framework Prevention Measures With HO-CDI Incidence Rate Trends, July 2019-March 2022 eFigure 1. Total Framework Intervention Implementation Score Over Time eFigure 2. Post Hoc Assessment of Study Power Loss due to COVID-19 eAppendix 1. Markdown Files from Statistical Analysis in R eAppendix 2. Supplemental Appendix Accessible via Open Science Framework Repository [file jamanetwopen-e243846-s001.pdf]

Supplemental Online Content

Turner NA, Krishnan J, Nelson A, et al. Assessment of CDC's hospital-onset *Clostridioides difficile* prevention framework in a regional hospital network. *JAMA Netw Open.* 2024;7(3):e243846. doi:10.1001/jamanetworkopen.2024.384

eTable 1. HO-CDI Prevention Tools and Interventions Deployed, Indexed by Framework Area

eTable 2. Overall Relative Changes in Core HO-CDI Prevention Areas by Intervention Site, July 2019-March 2022

eTable 3. Time Series Modeling of HO-CDI Incidence Between Intervention Sites and Control Sites With COVID-19 Effect (First Coprimary Analysis), July 2017-March 2022

eTable 4. Time Series Modeling of HO-CDI Incidence Between Intervention Sites and Control Sites Across a Range of Statistical Model Specifications and Structures (First Coprimary Analysis), July 2017-March 2022

eTable 5. Sensitivity Analysis of Second Coprimary Analysis (Pre- and Postintervention HO-CDI Trends Within Intervention Sites) Accounting for Potential COVID-19 Effect, July 2017-March 2022

eTable 6. Sensitivity Analysis of Second Coprimary Analysis (Pre- and Postintervention HO-CDI Trends Within Intervention Sites) Accounting for Varying Statistical Model Structures, July 2019-March 2022

eTable 7. Sensitivity Analysis of Second Coprimary Analysis (Pre- and Postintervention HO-CDI Trends Within Intervention Sites) Accounting for Potential COVID-19 Effect, July 2019-March 2022

eTable 8. Dose Effect of Framework Intervention Score

eTable 9. Association of Individual Framework Prevention Measures With HO-CDI Incidence Rate Trends, July 2019-March 2022

eFigure 1. Total Framework Intervention Implementation Score Over Time

eFigure 2. Post Hoc Assessment of Study Power Loss due to COVID-19

eAppendix 1. Markdown Files from Statistical Analysis in R

eAppendix 2. Supplemental Appendix Accessible via Open Science Framework Repository

This supplemental material has been provided by the authors to give readers additional information about their work.

eTable 1. HO-CDI Prevention Tools and Interventions Deployed, Indexed by Framework Area

CDI Prevention Framework Area	Resources and Interventions	Date Deployed
**Isolation and Contact Precautions**	1 Guide: Hand Hygiene	9/2019
	2 Tools/apps: Hand Hygiene & PPE Audit App	10/2019
	3 Blog: Educating Visitors on PPE Use	1/2020
	4 FAQ: Electronic Monitoring of Hand Hygiene	2/2020
	5 FAQ: Nurse-driven CDI Protocols	7/2020
	6 Dashboard example	6/2021
	7 Blog: Isolation of Asymptomatic Carriers	11/2021
**CDI Confirmation**	8 Guide: *C. difficile* Diagnostics (detailed version)	10/2019
	9 Guide: *C. difficile* Diagnostics (short version)	10/2019
	10 Rapid Review: Isolation of Asymptomatic Carriers	10/2019
	11 FAQ: Multistep *C. difficile* Testing	11/2019
	12 FAQ: Isolation Guidance for Asymptomatic Carriers	11/2019
	13 Blog: Whom to Test	1/2021
	14 *Blog: Clinical Decision Support System; How to encourage smart ordering?*	*4/2022*
**Environmental Cleaning**	15 Guide: EVS Cleaning and Auditing	8/2019
	16 Terminal Cleaning Checklist	8/2019
	17 EVS Supervisor Training	9/2019
	18 EVS Employee Training	9/2019
	19 Blog: Evidence for EVS	5/2021
	20 *Blog: C. difficile prevention and a Novel EVS Education Program and intervention plan*	7/2021
**Infrastructure**	21 Tools/apps: Mini Root Cause Analysis Review Tool	8/2019
	22 Tools/apps: Launch of Online CDI Dashboard	12/2019
	23 Tools/apps: (External Resource) PDSA Cycle Handout	1/2020
	24 Tools/apps: RCA Report Release	12/2020
	25 Blog: Data Visualization Tips	4/2021
	26 Data visualization article highlight	4/2021
	27 Blog: Cost Effectiveness of CDI Prevention Measures	4/2021
	28 Blog: Obstacles and Supports for Implementation	5/2021
**Antibiotic Stewardship**	29 Urinalysis stewardship toolkit	10/2019
	30 Blog: Ethnography of Antibiotic Decision Making	2/2020
	31 Blog: COVID and Stewardship	8/2020
	32 Blog: Empiric Therapy and COVID	8/2020
	33 Blog: Antibiotic Stewardship	4/2021
	34 Tools/apps: (External resource) AHQR Antibiotic Time Out Tool	5/2021
	35 *Blog: Asymptomatic Bacterisuputia*	*11/2021*
	36 *Blog: Microbiome Therapeutic to reduce recurrence of C difficile*	*1/2022*
	37 *Presentation: UTI: Opportunities for diagnostic Stewardship*	*3/2022*
	38 *Presentation: Pneumonia: Opportunities for stewardship*	*5/2022*

eTable 2. Overall Relative Changes in Core HO-CDI Prevention Areas by Intervention Site, July 2019-March 2022

	Isolation	Infrastructure	Confirmation	EVS	Stewardship	Total
Augusta	0/3 (0)	3/8 (0.38)	1/7 (0.14)	0/3 (0)	0/5 (0)	4/26 (0.15)
Carteret	0/3 (0)	0/8 (0)	3/7 (0.43)	2/3 (0.67)	2/5 (0.40)	7/26 (0.27)
Central Carolina	0/3 (0)	0/8 (0)	1/4 (0.25)	0/4 (0)	0/6 (0)	1/25 (0.04)
Chesapeake	1/3 (0.33)	1/8 (0.13)	1/5 (0.20)	0/3 (0)	2/6 (0.33)	5/25 (0.20)
Duke Raleigh	0/3 (0)	1/8 (0.13)	1/5 (0.20)	1/2 (0.50)	0/5 (0)	3/23 (0.13)
Duke Regional	2/3 (0.67)	0/8 (0)	1/5 (0.20)	0/1 (0)	0/6 (0)	3/23 (0.13)
Duke University	0/3 (0)	1/7 (0.14)	1/5 (0.20)	1/3 (0.33)	0/3 (0)	3/21 (0.14)
Frye	0/3 (0)	2/8 (0.25)	2/6 (0.33)	1/2 (0.50)	0/6 (0)	5/25 (0.20)
Iredell	1/3 (0.33)	0/8 (0)	1/5 (0.20)	0/3 (0)	0/6 (0)	2/25 (0.08)
Johnston	1/3 (0.33)	1/8 (0.13)	0/5 (0)	2/3 (0.67)	2/5 (.40)	6/24 (0.25)
Maria Parham	0/3 (0)	2/8 (0.25)	2/5 (0.40)	0/3 (0)	1/5 (0.20)	5/24 (0.21)
Nash	2/4 (0.50)	2/8 (0.25)	2/5 (0.40)	0/3 (0)	0/5 (0)	6/25 (0.24)
Princeton	1/4 (0.25)	1/8 (0.13)	1/8 (0.13)	0/5 (0)	0/5 (0)	3/30 (0.10)
Rex	0/5 (0)	2/8 (0.25)	3/7 (0.43)	0/1 (0)	0/5 (0)	5/26 (0.19)
Sarasota	1/4 (0.25)	2/8 (0.25)	0/7 (0)	0/1 (0)	0/5 (0)	3/25 (0.12)
Scotland	1/3 (0.33)	0/8 (0)	1/7 (0.14)	2/4 (0.50)	1/5 (0.20)	5/27 (0.19)
Southeastern	0/4 (0)	1/8 (0.13)	0/6 (0)	2/5 (0.40)	1/5 (0.20)	4/28 (0.14)
SOVAH	1/4 (0.25)	0/7 (0)	3/5 (0.60)	0/3 (0)	2/5 (0.40)	6/24 (0.25)
Wayne	0/4 (0)	0/8 (0)	1/8 (0.13)	0/4 (0)	0/5 (0)	1/29 (0.03)
Wilson	0/3 (0)	1/8 (0.13)	0/4 (0)	2/4 (0.50)	0/5 (0)	3/24 (0.13)

*Change reported relative to opportunity gaps present at each site (e.g., numerator = number of interventions undertaken during study period; denominator = number of prevention interventions not already implemented at study outset). Total potential prevention interventions per category are: 7 for isolation, 10 for infrastructure, 9 for confirmation, 7 for environmental services (EVS), 6 for stewardship, 39 in total.

eTable 3. Time Series Modeling of HO-CDI Incidence Between Intervention Sites and Control Sites With COVID-19 Effect (First Coprimary Analysis), July 2017-March 2022

Model structure:

HO-CDI = Time + Arm + Time x Arm + COVID + Time since COVID + Offset(patient days)

Parameter	IRR (95% CI)	p-value
Overall time trend	0.75 (0.56-1.01)	0.06
Arm, intervention vs control	2.78 (1.10-7.01)	0.03
Time x arm (test of slope change, intervention vs control)	0.80 (0.68-0.93)	<0.01
COVID level change	0.98 (0.76-1.26)	0.86
COVID trend change	1.36 (1.01-1.83)	0.04

*Slope/trend changes expressed per 12-month period

eTable 4. Time Series Modeling of HO-CDI Incidence Between Intervention Sites and Control Sites Across a Range of Statistical Model Specifications and Structures (First Coprimary Analysis), July 2017-March 2022

Model structure:

HO-CDI = Time + Arm + Time x Study Arm + Offset(patient days)

Model	Time	Arm	Time x Arm
GEE, ex	0.95 (0.89-1.03)	2.79 (1.10-7.05)	0.79 (0.67-0.94)
GEE, ind	0.90 (0.81-1.00)	2.44 (0.95-6.30)	0.83 (0.68-1.01)
ME, ri	0.95 (0.86-1.04)	3.14 (2.00-4.93)	0.79 (0.70-0.89)
ME, ri, zi	0.95 (0.86-1.04)	3.14 (2.00-4.93)	0.79 (0.70-0.89)
ME, rsri	1.02 (0.87-1.19)	3.00 (1.75-5.15)	0.82 (0.68-0.99)
ME, rsri, zi	1.02 (0.87-1.19)	3.00 (1.75-5.15)	0.82 (0.68-0.99)

eTable 5. Sensitivity Analysis of Second Coprimary Analysis (Pre- and Postintervention HO-CDI Trends Within Intervention Sites) Accounting for Potential COVID-19 Effect, July 2017-March 2022

Model structure:

HO-CDI = Time + Intervention (level change) + Intervention time (slope change) + COVID + Time since COVID + Offset(patient days)

Parameter	IRR (95% CI)	p-value
Baseline trend	0.71 (0.63-0.80)	<0.001
Level change	1.21 (0.88-1.64)	0.24
Slope change	1.19 (0.68-2.08)	0.55
COVID pandemic, level change	0.80 (0.52-1.22)	0.30
COVID pandemic, slope change	0.97 (0.58-1.64)	0.92

*Slope/trend changes expressed per 12-month period

eTable 6. Sensitivity Analysis of Second Coprimary Analysis (Pre- and Postintervention HO-CDI Trends Within Intervention Sites) Accounting for Varying Statistical Model Structures, July 2019-March 2022

Model structure:

HO-CDI = Time + Intervention (level change) + Intervention time (slope change) + Offset(patient days)

Model	Pre-intervention	Intervention, level	Intervention, slope
GEE, Ex	0.76 (0.68-0.85)	1.12 (0.89-1.42)	0.98 (0.77-1.24)
GEE, Ar	0.73 (0.64-0.84)	1.16 (0.81-1.65)	1.00 (0.77-1.29)
GEE, Ind	0.76 (0.68-0.85)	1.12 (0.88-1.42)	0.98 (0.77-1.24)
ME, RI	0.76 (0.70-0.82)	1.12 (0.98-1.30)	0.98 (0.88-1.09)
ME, RI, ZI	0.76 (0.70-0.82)	1.12 (0.98-1.29)	0.98 (0.88-1.09)
ME, RSRI	0.74 (0.67-0.83)	0.99 (0.98-1.00)	0.94 (0.84-1.04)
ME, RSRI, ZI	0.74 (0.67-0.83)	1.11 (0.96-1.27)	0.94 (0.84-1.04)

GEE = generalized estimating equation
ME = mixed effects
Ex = exchangeable correlation structure
Ar = autoregressive correlation structure
Ind = independent correlation structure
RI = random intercept
ZI = zero inflation
RSRI = random slope, random intercept

eTable 7. Sensitivity Analysis of Second Coprimary Analysis (Pre- and Postintervention HO-CDI Trends Within Intervention Sites) Accounting for Potential COVID-19 Effect, July 2019-March 2022

Model structure:

HO-CDI = Time + Intervention (level change) + Intervention time (slope change) + Offset(patient days)

Model	Lag (months)	Pre-intervention	Intervention, level	Intervention, slope
GEE	0	0.75 (0.67-0.83)	1.15 (0.92-1.42)	0.99 (0.78-1.26)
GEE	1	0.76 (0.68-0.85)	1.12 (0.88-1.42)	0.98 (0.77-1.24)
GEE	2	0.78 (0.70-0.87)	1.05 (0.86-1.28)	0.97 (0.77-1.22)
GEE	3	0.79 (0.70-0.88)	1.02 (0.81-1.28)	0.97 (0.77-1.22)

eTable 8. Dose Effect of Framework Intervention Score

Modeling Approach	Parameter	IRR (95% CI)
Intervention Score	Time (baseline trend)	0.81 (0.68-0.97)
	Total intervention score	0.95 (0.90-0.99)
Intervention Quintiles	Time x quintile (slope change test by quintile)	0.89 (0.83-0.95)

*Slope/trend changes expressed per 12-month period

eTable 9. Relative Impact of Individual Framework Prevention Measures on HO-CDI Incidence Rate Trends, July 2019-March 2022

General model structure (note that level and slope changes are included for each specific intervention listed below):

HO-CDI = Time + Intervention (level change) + Intervention time (slope change) + Offset(patient days)

Model	Parameter	Yearly IRR (95% CI)
Full model[a]	Baseline trend	1.03 (0.89-1.19)
	Isolation auditing, level	0.94 (0.74-1.19)
	Isolation auditing, slope	1.07 (0.91-1.26)
	Case reviews, level	1.25 (0.97-1.61)
	Case reviews, slope	0.90 (0.79-1.03)
	Two-step testing, level	0.54 (0.48-0.61)*
	EVS audits, level	1.33 (0.99-1.78)
	EVS audits, slope	1.06 (0.92-1.21)
	Terminal clean, level	1.68 (1.23-2.29)*
	Terminal clean, slope	0.82 (0.72-0.93)*
	Stewardship durations, level	1.01 (0.63-1.61)
	Stewardship durations, slope	0.89 (0.72-1.09)
	Stewardship, high yield, level	0.59 (0.47-0.76)*
	Stewardship, high yield, slope	0.87 (0.71-1.07)
	Stewardship, fluoroquinolone, level	0.48 (0.25-0.93)*
	Stewardship, fluoroquinolone, slope	1.04 (0.66-1.64)
Limited model[b]	Baseline trend	0.90 (0.79-1.02)
	Isolation auditing, slope	1.13 (0.94-1.34)
	Case reviews, slope	0.81 (0.68-0.96)*
	Two-step testing, level	0.50 (0.42-0.59)*
	EVS audits	1.12 (0.95-1.32)
	Stewardship, high yield	0.77 (0.60-0.99)*

[a]Full model included all prevention measures undertaken by at least 2 hospitals. Main model outcome was HO-CDI incidence.

[b]Limited model included only prevention measures undertaken by at least 2 hospitals with at least 6 months of time accrued before and after each intervention

*Delineates effect estimates with a 95% CI that does not cross 1.0

eFigure 1. Total Framework Intervention Implementation Score Over Time

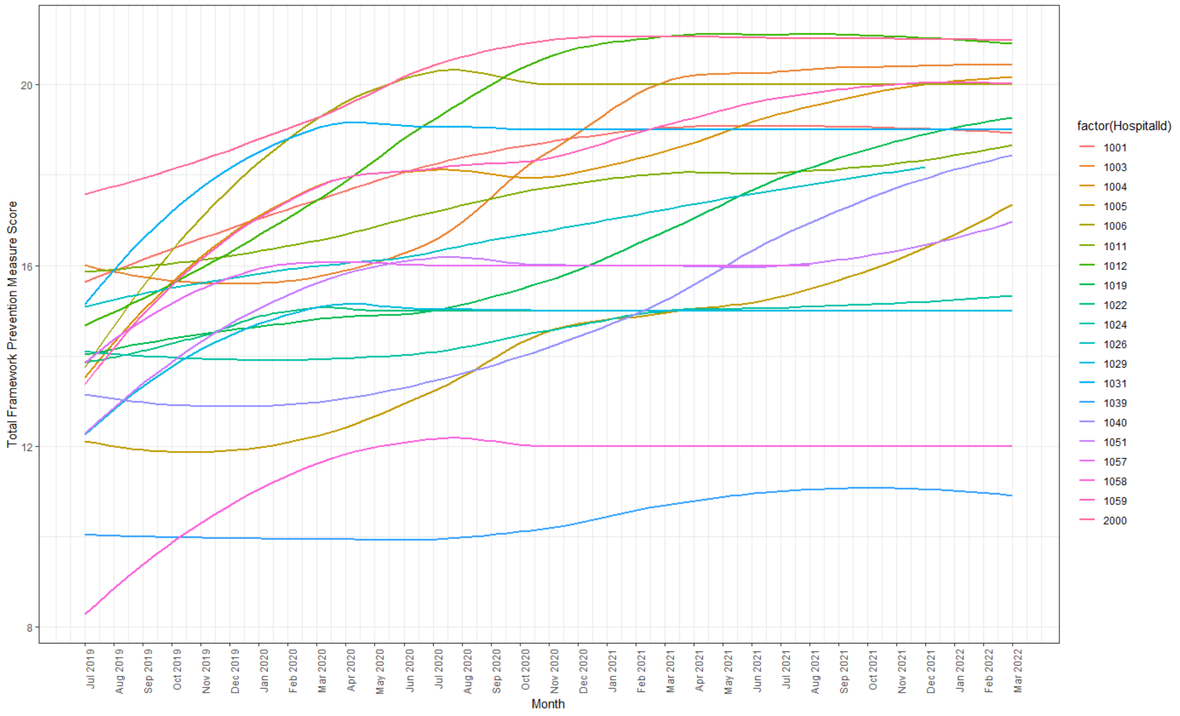

eFigure 2. Post Hoc Assessment of Study Power Loss due to COVID-19

To assess effect of COVID-19 pandemic on study power, we used pre-pandemic trends in intervention implementation (baseline slope trend from Figure 3 in main text) to estimate intervention implementation by study close in March 2022 assuming pre-pandemic trends were sustained. We then created a hypothetical data table in which sites' intervention rates increased in accordance with these prepandemic trends. We then used this hypothetical data table to provide inputs for the model associating intervention score with HO-CDI incidence rates (as in Table 2 in the main text).

The solid line represents modeled median HO-CDI rates across participating sites as observed in actual study. The dashed line represents modeled median HO-CDI rates across participating sites using:

1) Projected trends in interventions absent the COVID-19 pandemic (based on main manuscript, **Figure 3**)
2) Estimated intervention effects (based on main manuscript **Table 3**).

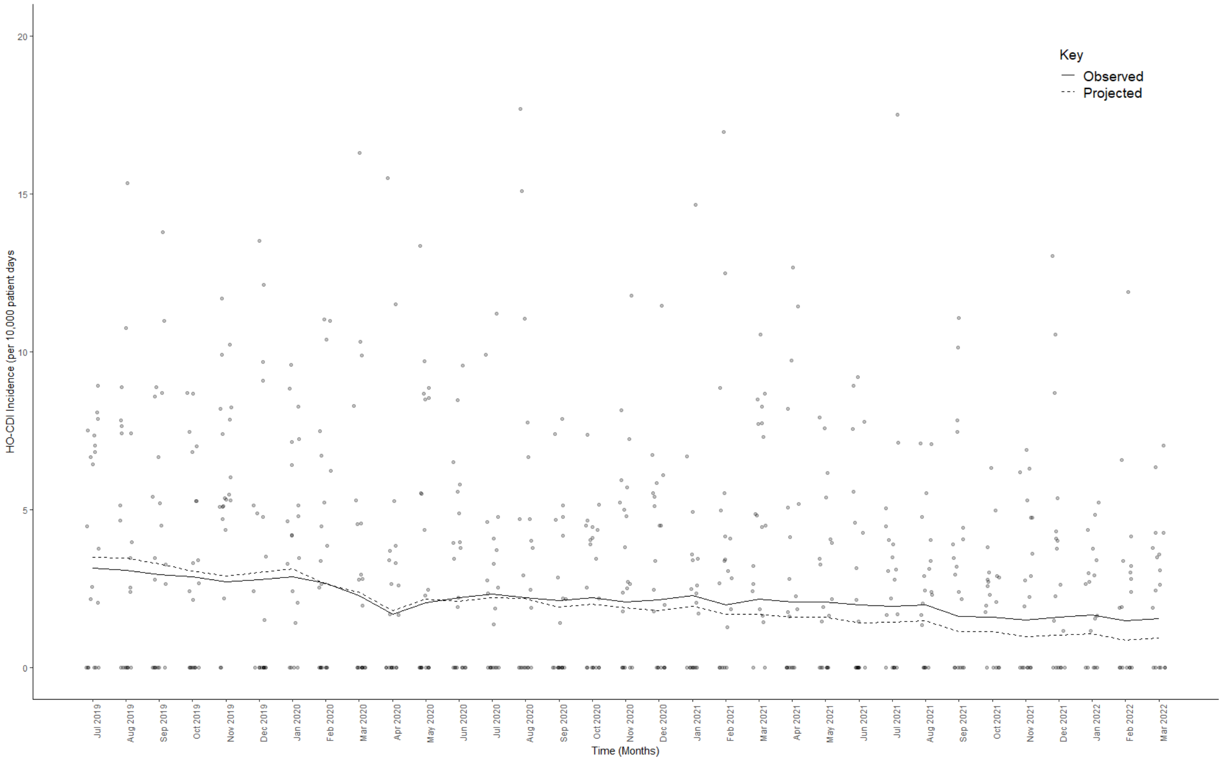

eAppendix. Markdown Files from Statistical Analysis in R

Analyst: Nicholas Turner, MD, MHSc
Edition: 27 June 2023

Barplot of Missingness by Variable:

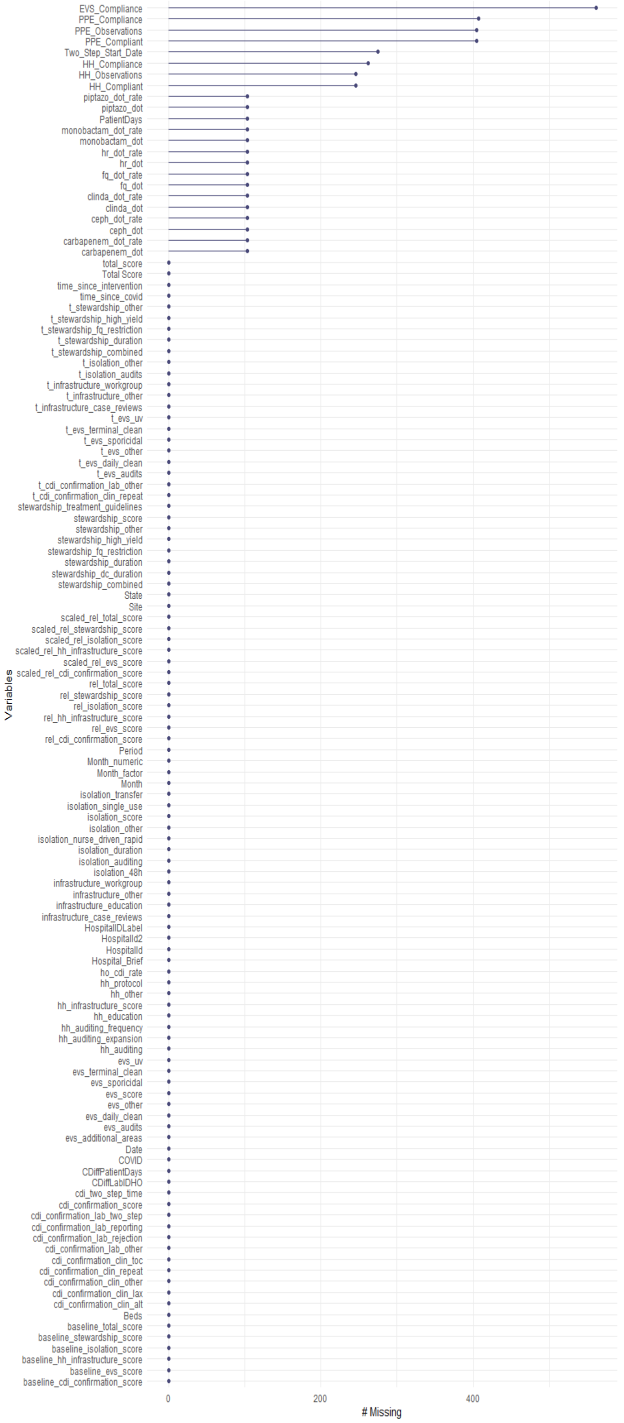

Heat map of missing data:

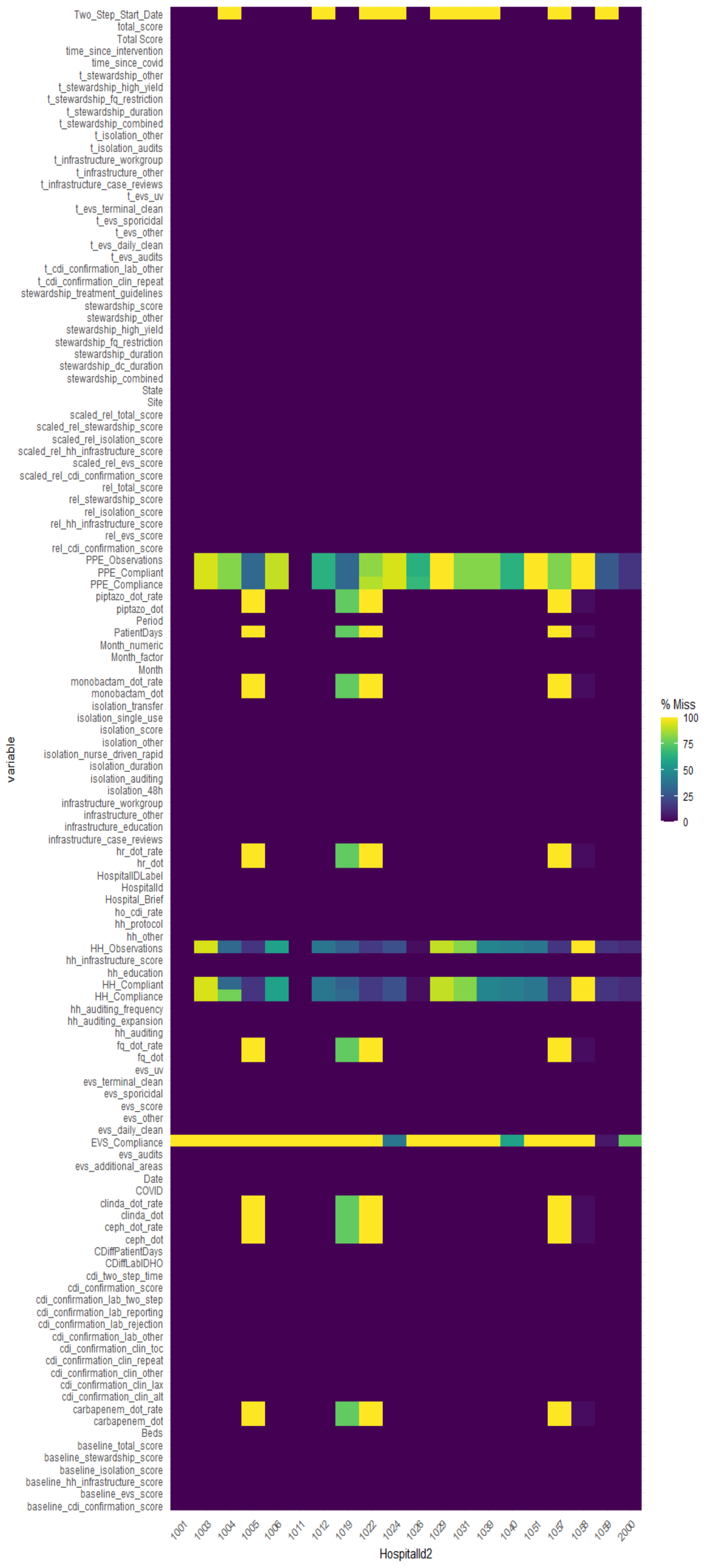

QQ Plot for Poisson Distribution Check

```
poisson <- fitdistr(cohort$ho_cdi_rate, "Poisson")
qqp(cohort$ho_cdi_rate, "pois", poisson$estimate, lambda=3.3)
```

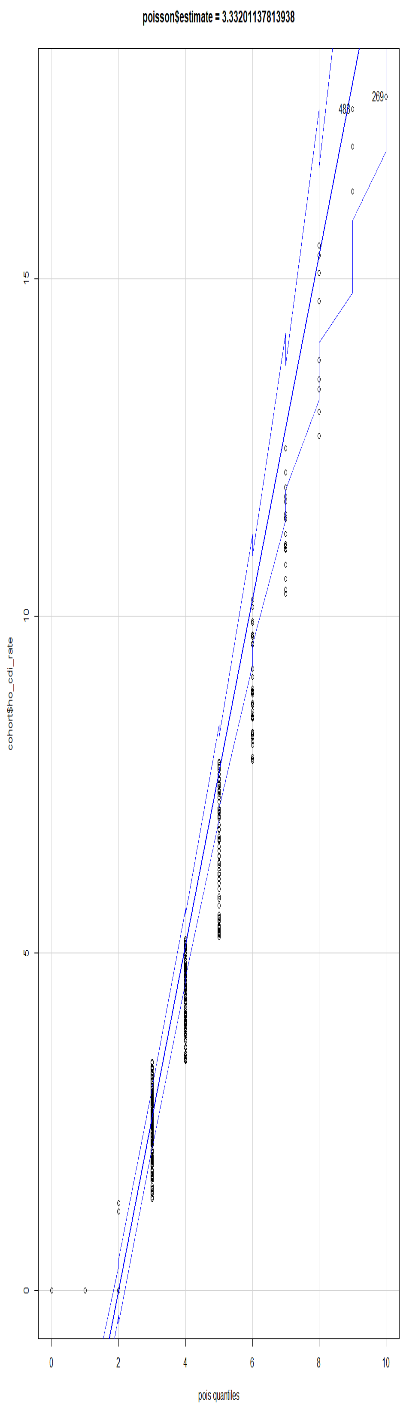

Primary Analysis

Coprimary analysis 1, contributing to Figure 1 in main text:

```
# GEE Modeling
gee_internal_ex <- geepack::geeglm(CDiffLabIDHO ~ month_numeric*Arm + offset(log(CDiffPat
ientDays)), id = factor(HospitalId), family="poisson", corstr = "exchangeable", waves=mon
th_numeric, data=cdi)

summary(gee_internal_ex)

##
## Call:
## geepack::geeglm(formula = CDiffLabIDHO ~ month_numeric * Arm +
##     offset(log(CDiffPatientDays)), family = "poisson", data = cdi,
##     id = factor(HospitalId), waves = month_numeric, corstr = "exchangeable")
##
##  Coefficients:
##                                Estimate   Std.err    Wald Pr(>|W|)
## (Intercept)                   -8.402737  0.442846 360.028  < 2e-16 ***
## month_numeric                 -0.003950  0.003194   1.529  0.21622
## ArmIntervention                1.024965  0.473421   4.687  0.03039 *
## month_numeric:ArmIntervention -0.019633  0.007157   7.526  0.00608 **
## ---
## Signif. codes:  0 '***' 0.001 '**' 0.01 '*' 0.05 '.' 0.1 ' ' 1
##
## Correlation structure = exchangeable
## Estimated Scale Parameters:
##
##             Estimate Std.err
## (Intercept)    1.904  0.2451
##   Link = identity
##
## Estimated Correlation Parameters:
##       Estimate Std.err
## alpha   0.4577  0.1031
## Number of clusters:   46  Maximum cluster size: 33

gee_internal_ex_outputs <- tidy(gee_internal_ex, conf.int=TRUE, conf.level=0.95) %>%
  mutate(IRR=exp(estimate),
         LL=exp(conf.low),
         UL=exp(conf.high),
         ann_IRR=exp(12*estimate),
         ann_LL=exp(12*conf.low),
         ann_UL=exp(12*conf.high))

kable(gee_internal_ex_outputs)
```

term	estimate	std.error	statistic	p.value	conf.low	conf.high	IRR	LL	UL	ann_IRR	ann_LL	ann_UL
(Intercept)	-8.4027	0.4428	360.028	0.0000	-9.2707	-7.5348	0.0002	0.0001	0.0005	0.000e+00	0.0000	0.000e+00
month_numeric	-0.0040	0.0032	1.529	0.2162	-0.0102	0.0023	0.9961	0.9898	1.0023	9.537e-01	0.8847	1.028e+00

term	estimate	std.error	statistic	p.value	conf.low	conf.high	IRR	LL	UL	ann_IRR	ann_LL	ann_UL
ArmIntervention	1.0250	0.4734	4.687	0.0304	0.0971	1.9529	2.7870	1.1019	7.0488	2.196e+05	3.2057	1.504e+10
month_numeric:ArmIntervention	-0.0196	0.0072	7.526	0.0061	-0.0337	-0.0056	0.9806	0.9669	0.9944	7.901e-01	0.6677	9.349e-01

Coprimary analysis 2, contributing to Figure 2

```
gee_internal_ind <- geepack::geeglm(CDiffLabIDHO ~ month_numeric + intervention + t_inter
vention + offset(log(CDiffPatientDays)), id = factor(HospitalId), family="poisson", corst
r = "independence", waves=month_numeric, data=cdi)

summary(gee_internal_ind)

##
## Call:
## geepack::geeglm(formula = CDiffLabIDHO ~ month_numeric + intervention +
##     t_intervention + offset(log(CDiffPatientDays)), family = "poisson",
##     data = cdi, id = factor(HospitalId), waves = month_numeric,
##     corstr = "independence")
##
##  Coefficients:
##                 Estimate  Std.err     Wald Pr(>|W|)
## (Intercept)    -6.99822  0.06962 10105.03  < 2e-16 ***
## month_numeric  -0.02297  0.00460    24.89  6.1e-07 ***
## intervention    0.11366  0.12178     0.87     0.35
## t_intervention -0.00176  0.01002     0.03     0.86
## ---
## Signif. codes:  0 '***' 0.001 '**' 0.01 '*' 0.05 '.' 0.1 ' ' 1
##
## Correlation structure = independence
## Estimated Scale Parameters:
##
##             Estimate Std.err
## (Intercept)      1.3   0.176
## Number of clusters:   20  Maximum cluster size: 57

gee_internal_outputs <- tidy(gee_internal_ind, conf.int=TRUE, conf.level=0.95) %>%
  mutate(IRR=exp(estimate),
         LL=exp(conf.low),
         UL=exp(conf.high),
         ann_IRR=exp(12*estimate),
         ann_LL=exp(12*conf.low),
         ann_UL=exp(12*conf.high))

kable(gee_internal_outputs)
```

term	estimate	std.error	statistic	p.value	conf.low	conf.high	IRR	LL	UL	ann_IRR	ann_LL	ann_UL
(Intercept)	-6.998	0.070	1.01e+04	0.000	-7.135	-6.862	0.001	0.001	0.001	0.000	0.000	0.000
month_numeric	-0.023	0.005	2.49e+01	0.000	-0.032	-0.014	0.977	0.969	0.986	0.759	0.681	0.846
intervention	0.114	0.122	8.71e-01	0.351	-0.125	0.352	1.120	0.882	1.422	3.912	0.223	68.596
t_intervention	-0.002	0.010	3.10e-02	0.860	-0.021	0.018	0.998	0.979	1.018	0.979	0.774	1.239

eAppendix 2. Supplemental Appendix Accessible via Open Science Framework Repository

https://doi.org/10.17605/OSF.IO/Z3SMV
