# Supplementary material for: CDC’s Hospital-Onset Clostridioides difficile Prevention Framework in a Regional Hospital Network
Source: JAMA Netw Open. 2024 Mar 27;7(3):e243846. doi: 10.1001/jamanetworkopen.2024.3846 (PMC10973890; doi:10.1001/jamanetworkopen.2024.3846)
Supplement: Supplement 2. — Data Sharing Statement [file jamanetwopen-e243846-s002.pdf]

Data Sharing Statement

Turner. Assessment of CDC's Hospital-Onset Clostridioides difficile Prevention Framework in a Regional Hospital Network. *JAMA Netw Open*. Published March 27, 2024. doi:10.1001/jamanetworkopen.2024.3846

Data
Data available: No

Additional Information
Explanation for why data not available: Markdown files including summary data can be made available upon request; not IRB approved to release any data which could include individual identifying information
